# Association between Low Serum Bicarbonate Concentrations and Cardiovascular Disease in Patients in the End-Stage of Renal Disease

**DOI:** 10.3390/diseases4040036

**Published:** 2016-11-15

**Authors:** Vaia D. Raikou, Despina Kyriaki

**Affiliations:** 1 1st Department of Medicine—Propaedaetic, School of Medicine, National & Kapodistrian University of Athens, Athens 11527, Greece; 2 Department of Nuclear Medicine, General Hospital “LAΪKO”, Athens 11527, Greece; dkyriaki@gmail.com

**Keywords:** hemodialysis, inflammation, metabolic acidosis, cardiovascular disease, lipoprotein oxidation, residual renal function

## Abstract

Background: Metabolic acidosis, a common condition particularly in the end-stage of renal disease patients, results in malnutrition, inflammation and oxidative stress. In this study, we focused on the association between low serum bicarbonate and cardiovascular disease in patients on intermittent dialysis. Methods: We studied 52 on-line-pre-dilution hemodiafiltration (on-l HDF) patients, 32 males and 20 females, with a mean age of 58.01 ± 15.4 years old. Metabolic acidosis was determined by serum bicarbonate concentrations less than 22 mmol/L. Residual renal function (RRF) was defined by interdialytic urine volume. Kaplan–Meier curves and Cox regression models were performed to predict coronary artery disease (CAD), defined by ejection fraction <50%, or diastolic dysfunction congestive heart failure (CHF) and peripheral vascular disease (PVD). Results: Kaplan–Meier analyses showed that a lower or higher than 22 mmol/L serum bicarbonate metabolic acidosis status was significantly associated with both PVD and diastolic dysfunction (log-rank = 5.07, *p* = 0.02 and log-rank = 5.84, *p* = 0.01, respectively). A similar prevalence of serum bicarbonate on CAD or CHF by low ejection fraction was not shown. The RRF was associated with PVD event and serum bicarbonate less than 22 mmol/L (log-rank = 5.49, *p* = 0.01 and log-rank = 3.9, *p* = 0.04, respectively). Cox regression analysis revealed that serum bicarbonate and RRF were significant risk factors for PVD after adjustment for confounders. Furthermore, RRF adjusted for covariates was shown to be a significant risk factor for diastolic dysfunction. Conclusion: Low serum bicarbonate was associated with peripheral vascular disease and diastolic dysfunction in intermittent dialysis. The residual renal function may impact patients’ outcomes through its relationship with metabolic acidosis status, particularly for peripheral vascular disease manifestation.

## 1. Introduction and Aim

Renal disease results in accelerated atherosclerosis and increased morbidity and mortality [1]. Even when the patients are undergoing renal replacement therapy, the mortality remains high, mainly for cardiovascular causes due either to uremia-related risk factors, such as anemia, hyperparathyroidism, inflammation, oxidative stress, hyperhomocysteinemia and malnutrition [2], or to traditional ones, such as age, male gender, diabetes, obesity, hypertension, smoking and dyslipidemia [3].

Cardiovascular disease is mainly characterized by vascular occlusive disease defined by myocardial infarction, cerebrovascular strokes and ischemic events of the limbs in these patients. The underlying disease that leads to these events progresses through various mechanisms, including left ventricular hypertrophy, plaque formation, arterial stiffening and endothelial dysfunction [4].

Metabolic acidosis, a common condition particularly in the end-stage of renal disease (ESRD) patients, leads tomalnutrition, inflammation, bone disease disorders and even higher death risk [5,6]. This is the result of a decreased ability to excrete nonvolatile acid and the reduced renal synthesis of bicarbonate. Despite the risks related to uncorrected metabolic acidosis, a high rate of hemodialysis patients has suboptimal correction of metabolic acidosis [7].

In this study, we focused on the association between low serum bicarbonate concentrations and cardiovascular disease in patients on intermittent dialysis.

## 2. Methods

### 2.1. Patients

This is a cross-sectional study of a cohort of 52 dialysis patients, 32 males and 20 females, with a mean age of 58.01 ± 15.4 years old. The data collection was performed during a time of 60 months, from 1 July 2007 until the end of June 2012.

Those with autoimmune diseases, infections, malignancy or other catabolic condition were excluded from our study. Furthermore, patients without regular vascular hemodialysis access and who had a dialysis catheter were not included in the study.

The treatment modality that was applied was on-line-pre-dilution hemodiafiltration (on-l HDF) for all patients. The median time on hemodialysis was 5.5 ± the interquartile range of 2.25–10.75 years.

The hemodialysis treatment was performed 3-times weekly with a dialysis time of 3.5–4 h per session, a filter of a 1.5–2 m^2^ surface area by high-flux synthetic membrane, defined by a ultrafiltration coefficient >20 mL/h [8] and a blood flow of 350–400 mL/min. A bicarbonate-based ultrapure buffer dialysis solution was used with a dialysate flow rate of 500–600 mL/min, a calcium concentration of 1.50–1.75 mmol/L, a sodium concentration of 138–145 mmol/L and low molecular weight heparin as anticoagulant therapy. The final concentration of bicarbonate in dialysate was 32 mmol/L. Dialysis dose defined by Kt/V/day for urea, which was calculated according to the formula of Daugirdas [9]. Patients were excluded if they had Kt/V for urea <1.2.

The enrolled patients were in a good status, on a free regular diet, and they did not have interdialytic peripheral edema, interdialytic orthostatic hypotension or other characteristics of an inaccurate dry body weight. However, patients with pre-dialysis blood pressure ≥140/90 (*n* = 19, a ratio of 36.5%) were considered hypertensive or if they were receiving anti-hypertensive drugs. Fifteen of the studied patients were current smokers (a ratio of 28.8%).

At the start of the study, cardiovascular disease was defined as the presence of coronary artery disease (CAD, *n* = 14, 26.9%), congestive heart failure (CHF) and peripheral vascular disease (PVD, *n* = 20, 38.5%). The coronary syndrome was documented by history of myocardial infarction, coronary artery angioplasty, bypass surgery or clinical signs of angina pectoris. Congestive heart failure was defined by systolic dysfunction (*n* = 11, 21.2%) or by the presence of diastolic dysfunction (*n* = 38, 73.1%). The existence of peripheral arterial disease was proven by both the measurement of the ankle-brachial blood pressure index (ABPI) and a set of clinical criteria, including cool skin, reduced or loss of ankle pulses on physical examination, symptomatic claudication, the presence of non-healing wounds or gangrene and by a history of past ischemic amputation or limb artery revascularization.

The first and the current cardiovascular events during the study were written down as one event for the studied cardiovascular manifestations.

Sixteen patients (30.7%) preserved a residual renal function (RRF), and they excreted up to 100 mL of interdialytic urine volume. Calcium channel blockers, beta-blockers or inhibitors of angiotensin II receptors were included in the receiving medications by our patients. Furthermore, 8 of the enrolled patients were receiving statin. Only calcium-free phosphate binders were prescribed, so 21 (40.3%) of our patients were receiving sevelamer carbonate using a small or regular dosage, 10 (19.2%) sevelamer hydrochloride, 8 (15.3%) lanthanum, 3 (5.7%) aluminum hydroxide, and 10 (19.2%) were not receiving any phosphate binders due to an acceptable serum phosphate concentration. None of our patients was receiving NaHCO_3_ per os. All of the studied patients wereon erythropoietin-α or-β therapy.

The underling renal diseases were hypertensive nephrosclerosis (*n* = 18, 34.6%), chronic glomerulonephritis (*n* = 15, 28.8%), polycystic kidney disease (*n* = 6, 11.5%), diabetic nephropathy (*n* = 5, 9.6%) and other/unknown (*n* = 8, 15.4%).

### 2.2. Approval and Consent

The study was approved by the ethics committee of the Hospitals “Laiko, University General Hospital of Athens” and Renal Unit of “Diagnostic and Therapeutic Center of Athens Hygeia SA”. Written informed consent was obtained from all subjects.

### 2.3. Blood Collection

In the study, patients’ blood was drawn just before the start of the mean weekly dialysis session in a twelve-hour fasting state from vascular access. Atthe end of the treatment, the blood pump speed was reduced to <80 mL/min, and blood samples were obtained at 2 min post-dialysis from the arterial dialysis tubing for the calculation of the adequacy of dialysis by Kt/V for urea.

Samples were centrifuged immediately; serum was separated and processed for various assays.

In each subject, three sequences of samples (every month within 3 months) were received for the serum bicarbonate measurements, and their average was used for statistical analysis, paying attention to the low serum bicarbonate level in combination with low arterial pH (acidemia) and decreased PCO_2_.

### 2.4. Laboratory Measurements

Albumin, calcium (Ca) corrected for the albumin levels, phosphate (P), high density lipoproteins (HDL) and low density lipoproteins (LDL) were measured by biochemical analysis, and hemoglobin values were also measured. The ratio of LDL/HDL was calculated.

High sensitivity C-reactive protein (hsCRP) and oxidized LDL (ox-LDL) serum concentrations were measured using enzyme linked immunoabsorbed assays (ΕLISA, Immundiagnostik AG, Germany and Immundiagnostik AG, Stubenwald-Allee, Bensheim, respectively) according to the manufacturer’s specifications.

The concentrations of intact-parathormone (i-PTH) were measured by radioimmunoassay (CIS bio international/France).

Metabolic acidosis was defined by serum bicarbonate concentrations less than 22.0 mmol/L, which were measured in a gas machine (Roche, combas b 121) by an electrode-based method taking care of the blood specimens [10].

The normalized protein catabolic rate for dry body mass (nPCR) was calculated from the urea generation rate [11]. The body mass index (BMI) was obtained from height and post-dialysis body weight.

### 2.5. Hemodynamic Measurements

Predialysis peripheral systolic and diastolic blood pressures (SBP and DBP, respectively) in enrolled patients were calculated as the mean of 10 measurements during a treatment month using an automatic sphygmomanometer OMRON M4-I (Kyoto, Japan). Mean peripheral pre-dialysis BP (MBP) was calculated as: MBP = DBP + 1/3 (SBP − DBP).

Electrocardiographic analysis and M-mode echocardiography were performed the day after dialysis with a Hewlett Packard SONOS 2500 using a 2.25-MHz transducer to estimate the systolic function, diastolic dysfunction and the ischemic findings according to the recommendations of the American Society of Echocardiography [12]. The systolic function of the left ventricle (LV) was assessed by the measurement of the ejection fraction (EF). The systolic weakness of LV is defined as EF < 50%. The diastolic function of LV is assessed by the determination of the maximum velocity of the early € and late (A) phase of ventricular filling and the calculation of the E/A ratio (ratio of early-to-late transmitral flow velocity). The diastolic dysfunction of LV is defined as E/A ≤ 1.

Furthermore, we measured the systolic pressure in both sides of the lower extremities, using a Doppler machine (Huntleigh Healthcare Ltd., Cardiff, UK).The ankle-brachial blood pressure index (ABPI) was calculated as the ratio of the lower values of ankle systolic pressure (pre- or post-tibial artery), divided by stabilized arm systolic pressure. ABPI values < 0.9 were rated as low, indicating peripheral vascular disease, and values up to 1.2 were rated as high.

### 2.6. Data Analysis

Data were analyzed using the SPSS 15.0 statistical package for Windows (SPSS Inc., Chicago, IL, USA) and expressed as the mean ± the standard deviation or as the median value (interquartile range) for data that showed a skewed distribution; differences between mean values were assessed by using unpaired *t*-test for two groups, and data that showed skewed distributions were compared with the Mann–Whitney *U*-test.

Correlations between variables were defined by the Pearson and Spearman coefficient, and *p*-values less than 0.05 were considered significant. Because of the duration of study being different in our data, the relationships between categorical variables were defined by log-rank tests with Kaplan–Meier analysis. We performed a Cox regression analysis to investigate serum bicarbonate concentrations as a possible independent predictor of coronary artery disease (CAD), congestive heart failure (CHF) and peripheral vascular disease (PVD) during a time of 60 months, after adjustment for the traditional and specific cardiovascular risk factors for dialysis patients, such as defined by interdialytic urine volume RRF, defined by Kt/V for urea dialysis adequacy, dyslipidemia and defined by i-PTH serum concentrations mineral bone disease. Furthermore, we examined the association between the RRF preservation or not and both serum bicarbonate concentrations less than 22 mmol/L and cardiovascular disease events by Kaplan–Meier curves. We built a model about the factors that could impact low serum bicarbonate (less than 22 mmol/L).

## 3. Results

**Figure 1:**
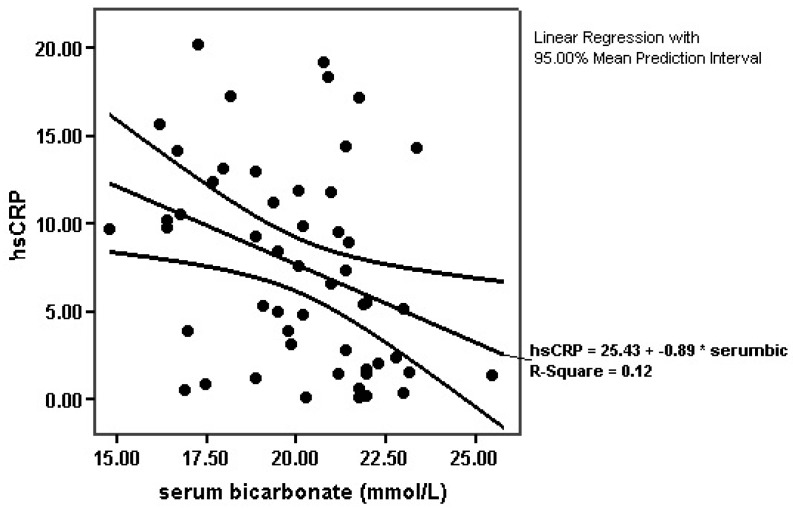
Association between serum bicarbonate levels and hsCRP (*r* = −0.384, *p* = 0.005).

Serum bicarbonate levels were inversely associated with hsCRP (*r* = −0.384, *p* = 0.005; Figure 1).

In Table 1, the differences between the groups of patients with serum bicarbonate levels more (*n* = 11) or less (*n* = 41) than 22 mmol/L are shown. We observed that the patients with serum bicarbonate levels less than 22 mmol/L were mainly males; they had a significantly higher age, hsCRP and i-PTH, although they had a significantly lower albumin and E/A ratio than the group of patients with serum bicarbonate levels greater than 22 mmol/L. The same group of patients had higher oxLDL serum concentrations and a higher ratio of CAD, CHF by low ejection fraction or diastolic dysfunction, PVD, hypertension and smoking. Regarding the distribution of preserved RRF in our data, we observed that in the group with serum bicarbonate levels less than 22 mmol/L, more patients did not have preserved RRF compared to those with RRF (*n* = 28, 68.3% versus *n* = 13, 31.7%).Additionally, most of the patients who were receiving sevelamer carbonate per os had serum bicarbonate less than 22 mmol/L, and all of our data with diabetes mellitus were included in this group of patients.

**Figure 2:**
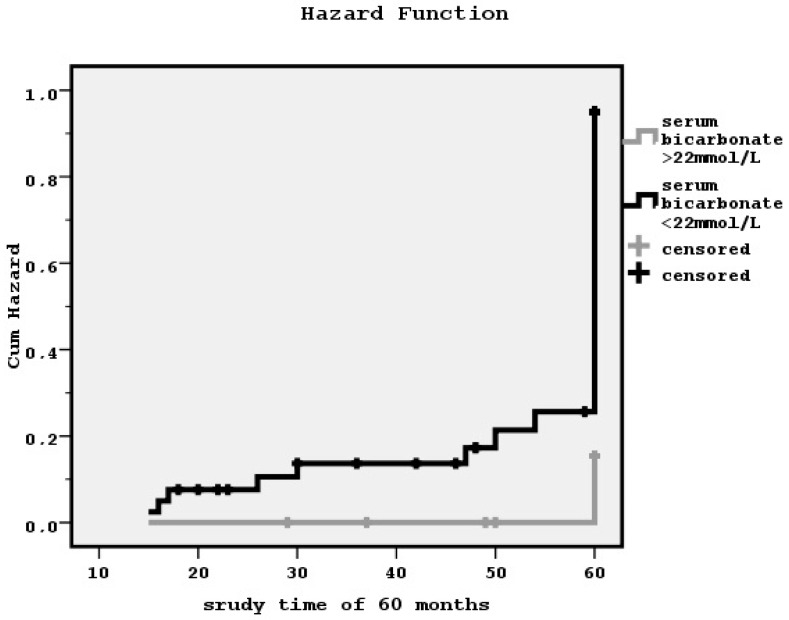
The influence of lower or higher than 22 mmol/L serum bicarbonate levels on peripheral vascular disease manifestation during a follow-up time of 60 months by the Kaplan–Meier curve (log-rank = 5.07, *p* = 0.02).

**Figure 3:**
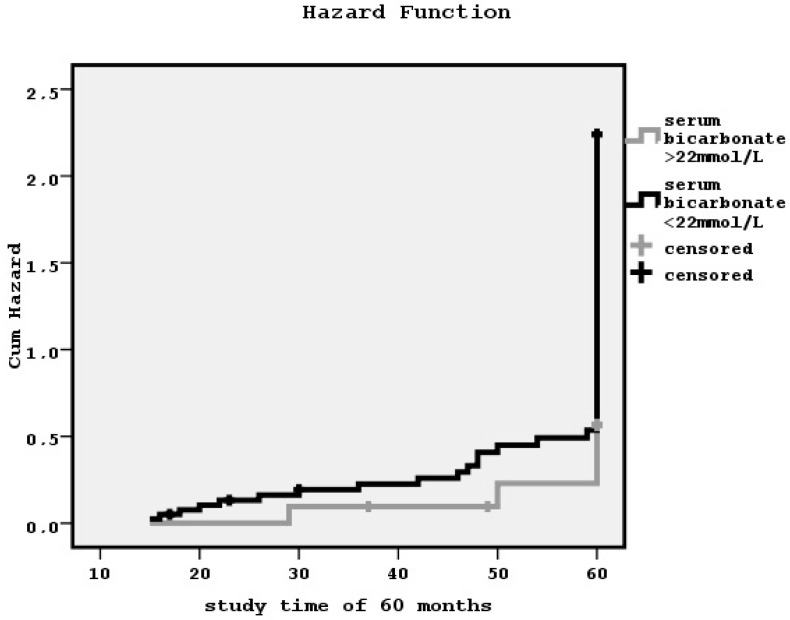
The impact of lower or higher than 22 mmol/L serum bicarbonate levels on diastolic dysfunction during a follow-up time of 60 months by the Kaplan–Meier curve (log-rank = 5.84, *p* = 0.01).

Kaplan–Meier analyses showed that metabolic acidosis status determined by lower or higher than 22 mmol/L serum bicarbonate levels was significantly associated with both PVD and defined by the existence of diastolic dysfunction CHF (log-rank = 5.07, *p* = 0.02 and log-rank = 5.84, *p* = 0.01, respectively; hazard functions in Figure 2 and Figure 3). The relationship between metabolic acidosis state and defined by low ejection fraction CHF or CAD was found to benon-significant.

**Figure 4:**
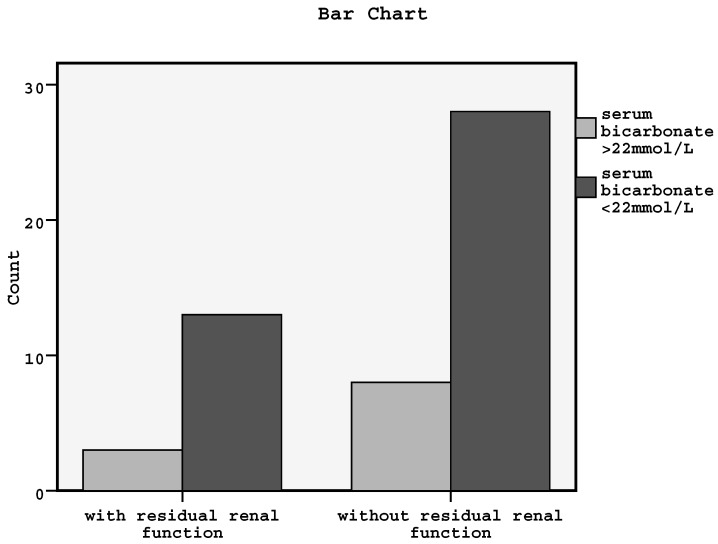
Bar chart for the association between residual renal function and metabolic acidosis status (log-rank = 3.9, *p* = 0.04).

Moreover, Kaplan–Meier curves showed that the relationship between the presence or not of RRF and both PVD manifestation and serum bicarbonate less than 22 mmol/L in our data was significant (log-rank = 5.49, *p* = 0.01 and log-rank = 3.9, *p* = 0.04, respectively) (the bar chart for the association between RRF and metabolic acidosis status is shown in Figure 4). However, the association between RRF and CAD, CHF by low ejection fraction or diastolic dysfunction was found to be non-significant.

Cox-regression analysis showed that serum bicarbonate levels and interdialytic urine volume were significant risk factors for PVD prevalence adjusted for defined by Kt/V for urea dialysis adequacy, dyslipidemia and defined by i-PTH serum concentrations mineral bone disease (Table 2). Furthermore, interdialytic urine volume was a significant risk factor for defined by diastolic dysfunction CHF after adjustment specific for these patients and traditional confounders (Table 3). Such a significance was not found for CAD, nor for defined by low ejection fraction CHF. In Table 4, variables that may influence the low serum bicarbonate are shown.

## 4. Discussion

End-stage renal disease (ESRD) results in atherogenic diathesis, which is mainly associated with oxidative stress, inflammation, malnutrition, hypertension and dyslipidemia [13,14]. This population of patients exhibits marked oxidative modification of lipids and lipoproteins, due mainly to the uremic environment and metabolic acidosis, usual conditions in these patients [15]. Furthermore, it has been already reported that metabolic acidosis promotes inflammation, releasing cytokines [16].

Indeed, in this study, we observed a significant positive association of metabolic acidosis status with hsCRP serum concentrations, as a definitely accepted marker of inflammation.

In a previous study, it has been suggested to maintain serum bicarbonate >22 mmol/L for all ESRD patients irrespective of dialysis modality, which was considered a complete correction of metabolic acidosis [17]. In ESRD patients, the low serum bicarbonate level must be considered in combination with the low arterial pH (acidemia) and decreased PCO_2_, which defines the existence of metabolic acidosis, rather than respiratory alkalosis, which is another clinical condition that causes decreased bicarbonate level, but without acidemia.

In the meantime, serum bicarbonate in these patients may be influenced by many variables, such as low or high dietary acid intake, malnutrition and catabolism, oral alkali intake (CaCO_3_ or NaHCO_3_), intake of sevelamer hydrochloride or sevelamer carbonate as a phosphate binder and the concentration of bicarbonate in the useddialysate during dialysis treatment.

In this study, we used the value of 22 mmol/L bicarbonate to divide our patients into two groups, and we carefully considered the low serum bicarbonate level in combination with low arterial pH (acidemia) and decreased PCO_2_. Regarding the variables that could influence the measured serum bicarbonate, we declare that the patients enrolled in the study were on a free regular diet, had no intensive catabolism or malnutrition, and most of the patients, who had serum bicarbonate less than 22 mmol/L, were receiving sevelamer carbonate per os as a phosphate binder and not sevelamer hydrochloride, which could cause a mild degree of metabolic acidosis. Furthermore, we used the same bicarbonate concentration in the dialysis dialysate in all of the patients enrolled in the study. Therefore, we could support that any of these factors significantly influenced the measured serum bicarbonate.

Comparing these two groups of patients, we noted that the patients with measured bicarbonate <22 mmol/L were males of an older age, had significantly higher hsCRP, i-PTH and oxLDL, lower albumin serum concentrations and a lower ratio of E/A as a marker of diastolic dysfunction, than the patients with serum bicarbonate >22 mmol/L. Furthermore, they had a higher ratio of CAD, CHF by low ejection fraction or diastolic dysfunction, PVD, hypertension and smoking than the group of patients with higher serum bicarbonate concentrations.

Moreover, we found that low bicarbonates were an independent significant risk factor for the manifestation of peripheral vascular disease adjusted for confounders. Furthermore, we found that metabolic acidosis status was significantly associated with the existence of both peripheral vascular disease and diastolic dysfunction. However, we did not find that the low bicarbonates were an independent risk factor for diastolic dysfunction after adjustment for covariates.

Diastolic dysfunction is a common finding in dialysis patients reflecting disorders in cardiac dilatation due mainly to the elevated stiffness of the myocardial wall in combination with fluid overload usually seen in these patients, despite the defined by ejection fraction systolic function being able to be preserved in a normal range [18].

On the other hand, peripheral vascular disease is an atherosclerotic disease that is frequently associated with coronary disease, and patients with PVD are at in increased risk of myocardiac infarction and vascular death [19].

In agreement, previous studies have reported that uncorrected metabolic acidosis leads to clinically significant consequences, like protein wasting, bone disease, morbidity, hospitalizations and even greater death risk [20,21,22,23] and that the correction of metabolic acidosis is one of the goals of adequate and effective dialysis [7].

The role of metabolic acidosis on vascular alterations has already been reported, as the mineral metabolism disturbances act through the existing metabolic acidosis in dialysis patients [24]. The influence of acidosis on vascular calcification is complicated, acting as a stimulator of the solubility of Ca × P products and as a blocker of phosphate uptake by the arterial smooth muscle cells, so acidosis may attenuate vascular calcification [25]. On the other hand, i-PTH, which is increased in the secondary hyperparathyroidism of renal disease, plays a crucial role in calcium homeostasis, and it may be a mediator of pathological calcification by both actions, preventing calcification in a dose-dependent manner by inhibiting alkaline phosphatase activity and promoting osteoblastic gene expression in bone [26,27]. The balance between stimulation and inhibition of calcification is disturbed in renal disease, and the final action of i-PTH is ectopic vascular calcification [28]. The ectopic vascular calcification by high i-PTH concentrations contributes to the manifestation of peripheral vascular disease in dialysis patients and to disorders in cardiac function. Additionally, the activation of lipid oxidation and inflammation by uncorrected metabolic acidosis is included in the factors that promote cardiovascular disease.

However, we found no significant association between low bicarbonate and systolic cardiac disorder or coronary artery disease. In agreement, a recent study showed no association of bicarbonate level with cardiovascular events and heart failure in patients with type 2 diabetes and nephropathy [29]. Furthermore, another previous study reported that the relationship between serum bicarbonate and congestive heart failure was nonlinear, and the risk of congestive heart failure was highest in participants with serum bicarbonate in the alkalotic range rather than in the acidosis state [30].

Interestingly, in this study, the existence of defined by interdialytic urine volume RRF was found as a significant independent factor for both PVD and diastolic dysfunction adjusted for confounders. Furthermore, the unadjusted association between the presence or not in RRF and PVD was shown to be significant in our data, but it was not significantly associated with coronary artery disease, low ejection fraction and diastolic dysfunction. On the other hand, the relationship between preserved or no RRF and low serum bicarbonate was also found to be significant, despite observing that most of our patients with preserved RRF were included in the group with serum bicarbonate levels less than 22 mmol/L, rather than in the group with serum bicarbonate greater than 22 mmol/L, due possibly to the high ratio of low bicarbonate in our data.

These findings could support that the RRF preservation in hemodialysis patients is associated with PVD and metabolic acidosis status, which, in the meantime, directly influences the demonstration of PVD. Consequently, the related preserved RRF low serum bicarbonate concentration may be the main factor that causes PVD manifestation or contributes to its exacerbation in these patients.

Regarding the role of low bicarbonate on diastolic dysfunction, our data may suggest an indirect relationship between them. However, the preservation of RRF affected by metabolic acidosis status may play a major role on diastolic cardiac dysfunction, even if we did not find a significant unadjusted association between RRF preservation and the existence of diastolic dysfunction. This finding may be attributed to our small amount of data, and bigger studies are need to clarify it.

The loss of RRF frequently reflects the severity of fluid overload in dialysis patients. In agreement, previously, it has been shown that patients with severe acidosis had less diuresis, and they presented important fluid overload and the need for increased ultrafiltration during the dialysis session, resulting in more intradialytic hypotension episodes [31]. These hemodynamic variations cause cardiovascular instability and determine alterations of vascular and myocardial walls, which in combination with arterial stiffness lead to the manifestation of diastolic dysfunction in these patients, even though the systolic function remains in the normal range [32].

A recent study has revived the importance of RRF preservation for better patient outcomes, including survival and quality of life [33]. Another previous study has suggested that residual renal function rather than overall adequacy (as estimated from total small solute removal rates) is an essential marker of patients’ outcomes in peritoneal dialysis [34]. Moreover, it has been already established that in peritoneal dialysis patients, the measured bicarbonate is significantly higher due mainly to a slower loss in RRF by peritoneal dialysis, suggesting that such a therapy provides a more complete correction of metabolic acidosis and possibly better outcomes than intermittent dialysis [34,35].

However, metabolic acidosis may be more complicated in hemodialysis than in peritoneal dialysis patients, due to the intervention of many factors, including the blood touching the dialysis membrane. In this study, our model showed that serum bicarbonate concentrations less than 22 mmol/L were significantly influenced by urine volume, serum albumin and dialysis vintage, adjusting forage, sex, diabetes mellitus, nPCR as a marker of catabolism and hsCRP as a marker of inflammation.

This study supports again the worldwide knowledge that corrected metabolic acidosis is associated with better biochemical and clinical outcomes in patients on hemodialysis. The preservation of RRF may be very beneficial for clinical outcomes of these patients, due partly to its relationship with metabolic acidosis status. However, such a relationship must be investigated by large randomized clinical trials in patients on intermittent dialysis.

## 5. Conclusions

This study showed that a low serum bicarbonate concentration was directly associated with peripheral vascular disease demonstration and indirectly with diastolic dysfunction in intermittent dialysis patients. Both relationships were found to be affected by preserved residual renal function. The underlying pathophysiology for the role of low bicarbonate on cardiovascular morbidity included enhanced inflammation, lipoprotein oxidation and bone disease.

## 6. Limitations

The limitation of our study is mainly the small number of included subjects. Furthermore, the data of arterial gas analyses, such as arterial pH and PCO_2_, were unavailable in our study.

## Figures and Tables

**Table 1:** Differences between groups of patients according to lower or higher than 22 mmol/L serum bicarbonate levels in a total of 52 patients enrolled in the study. i-PTH, intact-parathormone; MBP, Mean peripheral pre-dialysis BP; ABPI, ankle-brachial blood pressure index; EF, ejection fraction; E/A, ratio of early-to-late transmitral flow velocity.

	Patients with Serum Bicarbonate Less than 22 mmol/L (*n* = 41)	Patients with Serum Bicarbonate More than 22 mmol/L (*n* = 11)
Sex (males/females)	25 (61%)–16 (39%)	7 (63.6%)–4 (36.4%)
Age (years)	60.5 ± 14.5 *	48.7 ± 16
Dialysis vintage (years)	7.6 ± 7.1	7.4 ± 5.9
Kt/V for urea	1.4 ± 0.20	1.4 ± 0.23
nPCR (g/kg/day)	2.4 ± 0.54	2.4 ± 0.39
Urine volume (mL/day)	238.4 ± 166	190 ± 96.4
BMI (kg/m^2^)	24.5 ± 3.4	23.9 ± 2.13
Serum bicarbonate (mmol/L)	19.3 ± 1.9 *	22.8 ± 1.03
i-PTH (pg/mL)	14.4 ± 231.6 *	106.6 ± 94.7
Hb (gr/dL)	11.8 ± 1.3	12.3 ± 1.3
Albumin (gr/dL)	3.9 ± 0.2 *	4.15 ± 0.2
LDL/HDL	2.4 ± 0.8	2.6 ± 0.8
hsCRP (mg/L)	8.74 ± 5.7 *	3.32 ± 4.03
oxLDL (ng/mL)	140.7 ± 187.1	127.4 ± 112
MBP (mmHg)	97.1 ± 11.8	91.7 ± 11.2
ABPI	1.04 ± 0.4	1.01 ± 0.3
Diabetes mellitus (yes/no)	5 (12.2%)/36 (87.8%)	0 (0%)/11 (100%)
Hypertension (yes/no)	18 (44%)/23 (56.1%) *	1 (9%)/10 (91%)
Smoking (yes/no)	12 (29.3%)/29 (70.7%)	3 (27.3%)/8 (72.7%)
Coronary artery disease (yes/no)	12 (29.3%)/29 (70.7%)	2 (18.2%)/9 (81.8%)
Heart failure (EF<50%) (yes/no)	10 (24.4%)/31 (75.6%)	1 (9%)/10 (91%)
EF (%)	49.8 ± 14.8	56.6 ± 11.4
Diastolic dysfunction (yes/no)	34 (82.9%)/7 (17.1%) *	4 (36.4%)/7 (63.6%)
E/A ratio	0.98 ± 0.1 *	1.2 ± 0.2
Peripheral vascular disease (yes/no)	19 (46.3%)/22 (53.7%) *	1 (9%)/10 (91%)
Residual renal function (yes/no)	13 (31.7%)/28 (68.3%) *	3 (27.3%)/8 (72.7%)
Phosphate binders (yes/no)	34 (82.9%)/7 (17.1%)	8 (72.7%)/3 (27.3%) 5 (45.4%) 3 (27.2%)
Sevelamer carbonate	16 (39%)	
Sevelamer hydrochloride	7 (17.1%)	
Lanthanum	8 (19.5%)	
Aluminum hydroxide	3 (7.3%)	

* *p* < 0.05.

**Table 2:** Cox-regression analysis for the prevalence of serum bicarbonate levels and interdialytic urine volume on the manifestation of peripheral vascular disease.

	*p*-Value	Odds Ratio	Confidence Interval
Interdialytic urine volume	0.02	1.008	1.001–1.015
Kt/V for urea	0.8	0.35	0.009–2.95
LDL/HDL	0.14	0.20	0.02–1.76
i-PTH	0.17	0.99	0.98–1.003
Serum bicarbonate	0.03	0.45	0.20–0.96

**Table 3:** Cox-regression analysis for the prediction of diastolic dysfunction.

	*p*-Value	Odds Ratio	Confidence Interval
Age	0.8	0.99	0.9–1.06
Family atherosclerotic history	0.5	1.9	0.2–16.9
Previous atherosclerotic events	0.07	10.8	0.7–149.7
Cardioactive medications	0.3	2.3	0.4–11.9
Smoking	0.5	0.4	0.03–6.3
Urine volume	0.02	1.01	1–1.02
LDL/HDL	0.8	1.12	0.4–3.04
i-PTH	0.05	1.008	1–1.02
Serum bicarbonate	0.4	1.3	0.6–2.8

**Table 4:** Cox-regression analysis for the role of variables on serum bicarbonate concentrations less than 22 mmol/L.

	*p*-Value	Odds Ratio	Confidence Interval
Age	0.1	1.04	0.9–1.1
Sex	0.3	0.47	0.09–2.2
Diabetes mellitus	0.6	0.3	0.006–22.1
Dialysis vintage	0.03	1.3	1.02–1.7
Urine volume	0.02	1.007	1.001–1.02
nPCR	0.4	0.5	0.1–2.37
Serum albumin	0.03	0.005	0–0.7
hsCRP	0.7	1.04	0.8–1.3
